# Comparative Efficacy and Safety of Immunotherapy Alone and in Combination With Chemotherapy for Advanced Non-small Cell Lung Cancer

**DOI:** 10.3389/fonc.2021.611012

**Published:** 2021-03-18

**Authors:** Xue Wang, Xiaomin Niu, Na An, Yile Sun, Zhiwei Chen

**Affiliations:** Department of Shanghai Lung Cancer Center, Shanghai Chest Hospital, Shanghai Jiao Tong University, Shanghai, China

**Keywords:** immunotherapy, chemotherapy, immune-related adverse event, NSCLC, PD-L1

## Abstract

There is a lack of direct cross-comparison studies in clinical trials between immunotherapy alone and combination treatment, especially in Non-Small Cell Lung Cancer (NSCLC) patients with high PD-L1 expression. To determine if anti-PD-(L)1 antibody combined with chemotherapy is more efficient than immune checkpoint inhibitor (ICI) monotherapy for advanced NSCLC patients in the real-world data. We retrospectively collected 325 patients with advanced NSCLC treated with ICI alone with or without chemotherapy from 11th July 2016 to 26th May 2020 to investigate which treatment scenario is the most efficient, and how clinical factors impact response. Patients with advanced NSCLC were treated with ICI monotherapy (178/325, 54.8%) or in combination with chemotherapy (147/325, 45.2%). The objective response rate and disease control rate were higher in the combination group than the monotherapy group. Patients (including those with distant metastasis) treated with chemo-immunotherapy were associated with a significantly longer median PFS and OS compared with the monotherapy group, irrespective of the PD-L1 expression level and previous treatment lines. No significant increase in the risk of immune-related adverse events (irAEs) was found after combination with chemotherapy (50.6 vs. 57.8%). IrAEs predicted better PFS of immunotherapy in the monotherapy group, especially for patients with late irAEs (after ≥4 cycles). Collectively, we demonstrated that ICI monotherapy plus chemotherapy might have better anti-tumor activity and an acceptable side-effect profile regardless of PD-L1 level or previous treatment lines. Both regimens were well-tolerated and cost-effective, the more efficient is usually recommended.

## Introduction

The advent of immune checkpoint inhibitors (ICIs) has radically changed the therapy paradigm in advanced NSCLC over the past 5 years. A remarkable improvement in the management of metastatic NSCLC occurred in 2015, when nivolumab was approved for the treatment of patients with progressive disease during or after a platinum-doublet treatment (1). Both anti-programmed death 1 (PD-1) and anti- programmed death ligand-1 (PD-L1) antibodies have demonstrated their benefits in comparison with standard chemotherapy (2–5).

Due to the encouraging results from clinical trials, the U.S. Food and Drug Administration (FDA) granted approval for ICIs as monotherapy in advanced NSCLC. What's more, pembrolizumab is recommended as the first-line treatment in oncogene-negative tumors with high (Tumor Proportion Score, TPS ≥50%; category 1) or low PD-L1 expression (1% ≤ TPS <50%; category 2B); and atezolizumab or pembrolizumab combined with carboplatin-based doublet as the front-line treatment is also approved (category 1) (6). Chemotherapy or immunotherapy alone (no previous ICI treatment) is preferred as the second-line treatment for PS 0–2. Nevertheless, deciding between therapeutics remains a challenge today.

The objective of this retrospective study is to investigate the efficacy and safety of ICI monotherapy or in combination with chemotherapy for advanced NSCLC patients in the real-world.

## Materials and Methods

### Participants

325 patients had stage IIIB-IV NSCLC were retrospectively included from Shanghai Chest Hospital from 11th July 2016 to 26th May 2020; measurable disease on the basis of the Response Evaluation Criteria in Solid Tumors version 1.1 (RECIST v1.1); a baseline Eastern Cooperative Oncology Group performance status (ECOG PS) of 1; and all stage IIIB patients were not suitable for radiotherapy (*n* = 36). Baseline distant metastases were ascertained by CT scans or MRI with contrast imaging.

32/37 (86.4%) patient were epidermal growth factor receptor (EGFR) sensitizing mutations (exon 19 deletion, exon 21 L858R, L861Q or L861R, exon 20 S786I or T790M mutations), and 28/32 (87.5%) patients had progressive disease or intolerance to treatment with approved first-, second-, and/or third-generation EGFR-TKIs. 2/32 (6.25%) patients with exon 21L858R mutation were treatment-naïve; and another 2/32 (6.25%) received chemotherapy. Nobody had anaplastic lymphoma kinase (ALK) translocations. PD-L1 expression was analyzed by immunohistochemistry assay in archival or freshly collected tumor tissue with different antibodies [5/325 (1.54%) were 22C3, 20/325 (6.15%) were SP263, 86/325 (26.46%) were E1L3N, and 6/325 (1.85%) were 28-8]. Histologic slides with a minimum of 100 tumor cells were required for PD-L1 assessment.

### Treatments

Patients were treated with anti-PD-(L)1 alone (*n* = 178) or combined with chemotherapy (*n* = 147), and it was their first exposure to ICIs. The dosage of drugs administered are shown in Supplementary Table 1. 281/325 (86.46%) patients received anti-PD-1 antibody treatment [71/281 (25.27%) combined with pemetrexed and carboplatin, 19/281 (6.76%) with paclitaxel and carboplatin, 15/281 (5.34%) with nab-paclitaxel and carboplatin, 23/281 (8.19%) with other chemotherapeutics]; 44/325 (13.54%) received anti-PD-L1 antibody treatment [19/44 (43.18%) combined with pemetrexed and carboplatin].

Treatment was given until disease progression, severe toxicity, or death. Assessments of progression occurred every two cycles until disease progression as per RECIST v1.1 (tumor assessments of nivolumab occurred every three cycles).

### Outcomes

All patients were followed up for survival until death, or loss-to follow-up (4/325, 1.2%) from 11th July 2016 to 26th May 2020. Progression-free survival (PFS) and Overall survival (OS) were measured as the time between start of treatment and documented disease progression or death owing to any cause (PFS) or to the latter (OS). Time to treatment failure (TTF) was assessed from immunotherapy to cessation of ICI treatment for any reason. Disease control rate (DCR) refers to the proportion of patients with complete response (CR), partial response (PR) or stable disease (SD) for at least 6 months. Objective response rate (ORR) was defined as the proportion of patients with CR or PR for at least 6 months. Duration of response (DOR), defined as initial CR or PR to progressive disease (PD) or death.

Adverse events (AEs) were graded according to the National Cancer Institute Common Terminology Criteria for Adverse Events (NCI-CTCAE), version 4, and were classified according to their characteristics: treatment-related AEs (trAEs) and immunotherapy-related AEs (irAEs) (7–10). Assessments were done by at least three independent medical professionals.

In addition, progression in no-target lesions was quantified based on four progression items: pre-existing lesions, new intrathoracic metastasis, new extrathoracic metastasis, or new malignant effusion (11, 12). Score 1 point for each progression item and add up the total. The final score will show the level of tumor burden.

### Statistical Analyses

Associations between variables and PFS or OS were analyzed using Kaplan-Meier survival curves, the log-rank test, and univariate or multivariate Cox regression models. Multivariate hazard ratios (HRs) were estimated with a stratified Cox regression model, and 95% confidence Intervals (CIs) were calculated with the Brookmeyer-Crowley method. Subgroup analyses were done with unstratified HRs estimated from a cox proportional hazards model. Analyses were carried out using IBM SPSS Statistics 21.0 software. Categorical variables were compared in the same platform by the Fisher's exact or chi-square test. A two-tailed *p* < 0.05 was considered significant.

## Results

### Clinicopathologic Features and Outcomes

**Figure 1:**
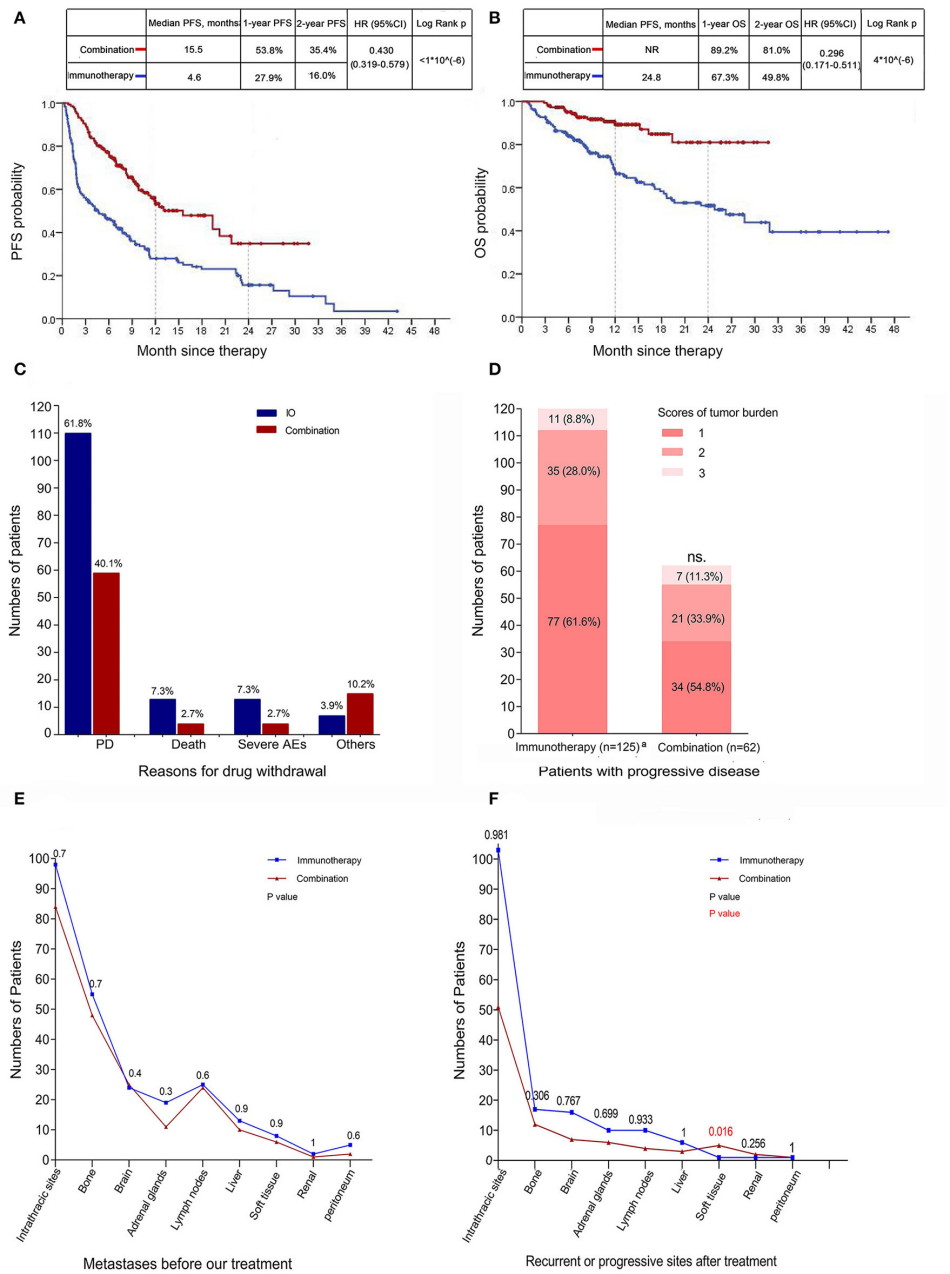
Outcomes of advanced lung cancer patients treated with ICI alone or in combination with chemotherapy. **(A,B)** PFS and OS in tumors treated with immunotherapy alone (*n* = 178) or in combination with chemotherapeutics (*n* = 147) [HR 0.430, 95% CI 0.319–0.579, log-rank *p* <1*10^(−6)^]. **(C)** Reasons for drug withdrawal in tumors from patients received immunotherapy or combination therapy. **(D)** Tumor burden scores of patients with progressive disease in different group (*P* = 0.284). **(E,F)** Metastases before our treatment **(E)** and recurrent or progressive sites after immunotherapy **(F)** were shown. Statistical analysis for Kaplan-Meier plots used the log-rank test and statistical analysis for progressive sites used Chi-square test; tumor burden score was tested by Independent samples *t*-test. PFS, progression-free survival; OS, overall survival; HR, hazard ratio; CI, confidence interval. ^a^Information on disease progression in two patients was not available.

178/325 (54.8%) patients were administered a single ICI, whereas the remainder of patients were combined with chemotherapy (147/325, 45.2%). The baseline characteristics are demonstrated in Table 1. Immunotherapy group had a significantly lower ORR in comparison with combination group (27 of 178, 15.2% vs. 64 of 147, 43.5%, respectively), similar results were obtained in DCR (72 of 178, 40.4% vs. 100 of 147, 68.0%, respectively). The median DOR was 18.9 months (95% CI: NR) with combination group and 21.5 months (95% CI: 12.2–30.7) with immunotherapy group; 50 (69.4%) of 72 patients in the combination group and 15 (48.4%) of 31 patients in the immunotherapy group had an ongoing response at the time of data cutoff (Table 2). PFS was significantly reduced in monotherapy arm [(combination vs. immunotherapy) HR: 0.430, 95% CI: 0.319–0.579, log-rank *p* <1^*^10^(−6)^] (Figure 1A). The median OS for combination treatment has not been reached [(combination vs. immunotherapy) HR: 0.296, 95% CI: 0.171–0.511, log-rank *P* = 4^*^10^(−6)^] (Figure 1B). Reasons for drug withdrawal are mainly progression disease (Figure 1C). However, there was no significant difference in the scores of tumor burden between them (*P* = 0.284; Figure 1D; Table 2). The predominant sites for disease progression after ICIs were shown in Figure 1F, there was no significant difference between those two groups except soft tissue (*P* = 0.016) (baseline metastases were demonstrated in Figure 1E, no significant difference was found).

**Table 1A:** Population characteristics.

**Characteristic**	**Immunotherapy (*N* = 178)**	**Combination (*N* = 147)**	***P*-value**
Age (mean ± SD, y)	63.3 ± 8.5	60.9 ± 8.9	0.013
ECOG PS, *n* (%)			
1	178 (100.0)	147 (100.0)	
Gender, n (%)			
Male	147 (82.6)	113 (76.9)	0.200
Female	31 (17.4)	34 (23.1)	
BMI, *n* (%)			
<18.5, underweight	17 (9.6)	4 (2.7)	0.674
18.5–22.9, normal	64 (36.0)	65 (44.1)	
23.0–24.9, overweight	44 (24.7)	37 (25.2)	
≥25, obesity	53 (29.8)	41 (27.9)	
Smoking status, *n* (%)			
Never-smoker	54 (30.3)	52 (35.4)	0.335
Former/active smoker	124 (69.4)	95 (64.6)	
Pack-year of smoking, *n* (%)^a^			
<20	14 (7.9)	15 (10.2)	
20– <40	40 (22.5)	35 (23.8)	0.117
≥40	70 (39.3)	45 (30.6)	
Tumor histology, *n* (%)			
Adenocarcinoma	98 (55.1)	101 (68.7)	0.015
Squamous carcinoma	60 (33.7)	35 (23.8)	
NSCLC	9 (5.1)	1 (0.7)	
Others^b^	11 (6.2)	10 (6.8)	
Metastatic sites, *n* (%)			
Bone	55 (30.9)	48 (32.7)	0.742
Lung/pleura	98 (55.1)	84 (57.1)	
Brain	24 (13.5)	25 (17.0)	
Distant lymph nodes	25 (14.0)	24 (16.3)	
Adrenal glands	19 (10.7)	11 (7.5)	
Liver	13 (7.3)	10 (6.8)	
Others^c^	18 (10.1)	9 (6.1)	
EGFR, *n* (%)			
Mutation^d^	23 (12.9)	14 (9.5)	0.004
Wild-type	132 (74.2)	128 (87.1)	
Unknown	23 (12.4)	5 (3.4)	
PD-L1, *n* (%)			
Negative or <25%	34 (19.1)	37 (25.2)	0.416
≥25%	33 (8.5)	26 (17.7)	
Unknown	111 (62.4)	84 (57.1)	

a Packs per day × years smoked in ever smokers;

b *Immunotherapy: 2 neuroendocrine tumors, 1 severe dysplasia, 1 sarcomatoid carcinoma, 1 adenosquamous carcinoma, 6 malignant tumors; Combination: 2 lymphoepithelioma-like carcinomas, 3 adenosquamous tumors, 4 malignant tumors, 1 neuroendocrine carcinoma*.

c *Immunotherapy: 8 soft tissues, 5 peritoneum, 3 pancreases, 2 kidneys; Combination: 6 soft tissues, 2 peritoneum, 1 kidney*.

d Immunotherapy: 19 del (5 cases), 21 L858R (10 cases), 21 L858R and 20 S786I (1 case), EGFR 20 T790M and S786I (1 case), 20 S786I (1 case), 21 L861Q (1 case), 21 L861R (1 case), 21 L858R and 20 T790M (1 case), and non-sensitive EGFR mutations (2 cases);

*Combination: 19 del (4 cases), 21 L858R (3 cases), 21 L858R and 20 S786I (1 case), 21 L858R and 20 T790M (1 case), 19 del and 20 T790M (2 cases), and non-sensitive EGFR mutations (3 cases)*.

*PS, Performance Status; SD, standard deviation; BMI, body mass index; PD-1, programmed cell death-1; PD-L1, programmed cell death ligand 1; IO, immunotherapy alone; Chemo, chemotherapy*.

**Table 1B:** Characteristics of treatment regimens.

**Characteristic, *N* (%)**	**Immunotherapy (*N* = 178)**	**Combination (*N* = 147)**	***P*-value**
Previous treatment lines			
None	33 (18.5)	114 (77.6)	<1*10^∧^(−6)
1L	109 (61.2)	19 (12.9)	
≥2L	36 (20.2)	14 (9.5)	
ICI			
Nivolumab	98 (55.1)	9 (6.1)	<1*10^∧^(−6)
Pembrolizumab	25 (14.0)	50 (34.0)	
Tislelizumab	11 (6.2)	46 (31.3)	
Sintilimab	15 (8.4)	18 (12.2)	
Toripalimab	4 (2.2)	4 (2.7)	
Camerelizumab	0 (0)	1 (0.7)	
Atezolizumab	5 (2.8)	19 (12.9)	
Durvalumab	20 (11.2)	0 (0)	
Target of ICI			
PD-1	153 (86.0)	128 (87.1)	0.769
PD-L1	25 (14.0)	19 (12.9)	
Chemotherapeutic			
Pemetrexed		90 (60.5)	
Paclitaxel		19 (12.9)	
Nab-Paclitaxel		15 (10.2)	
Docetaxel		14 (9.5)	
Gemcitabine		5 (3.4)	
Others^a^		4 (2.7)	
Maintenance treatment			
IO+Chemo		60 (40.8)	
IO		32 (21.8)	
Chemo		7 (4.8)	

a *Three patients received vinorelbine therapies; 1 patient received etoposide therapy*.

**Table 2:** Treatment efficacy results.

**Characteristic**	**Immunotherapy**	**Combination**	***P*-value**
	**(*N* = 178)**	**(*N* = 147)**	
Best response, *n* (%)			
Partial response	31 (17.4)	72 (49.0)	<1*10^∧^(−6)
Stable disease	75 (42.1)	62 (42.2)	
Progressive disease	72 (40.4)	13 (8.8)	
ORR^a^, *n* (%) [95% CI]	27 (15.2) [9.8–20.5]	64 (43.5) [35.4–51.6]	<1*10^∧^(−6)
DCR^b^, *n* (%) [95% CI]	72 (40.4) [33.2–47.7]	100 (68.0) [60.4–75.7]	1*10^∧^(−6)
DOR, months, median (95% CI)	21.5 (12.2–30.7)	18.9 (NR-NR)	0.803
TTF, months, median (95% CI)	3.6 (1.5–5.8)	9.8 (7.5–12.1)	1.2*10^∧^(−5)
PFS, months, median (95% CI)	4.6 (2.1–7.1)	15.5 (9.8–21.3)	<1*10^∧^(−6)
OS, months, median (95% CI)	24.8 (16.3–33.3)	NR (NR-NR)	4*10^∧^(−6)
Scores of tumor burden, mean ± SD	1.45 ± 0.67	1.56 ± 0.70	0.284

a The proportion of patients with CR or PR for at least 6 months;

b The proportion of patients with CR or PR or SD for at least 6 months;

*ORR, objective response rate; DCR, disease control rate; DOR, duration of response; TTF, time to failure; PFS, progression-free survival; OS, overall survival*.

Subgroup analyses revealed that almost all subgroups were significantly associated with improved PFS in combination group, except for EGFR mutation, previous treatment line = 1 and baseline liver, adrenal gland, or lymph node metastasis (Supplementary Figure 1A). Similar results were demonstrated in OS, whereas patients with brain, liver, distant lymph node or adrenal gland metastases, 1 or ≥2 previous treatment lines, PD-L1 TPS expression <25%, EGFR mutation and overweight were not associated with better OS in the combination group (Supplementary Figure 1B). Furthermore, multivariate analysis demonstrated that the PD-L1 ≥25% or unknown, 0, 1, or ≥2 previous treatment lines and obesity were related with better PFS or OS of patients with advanced-stage NSCLC (Supplementary Tables 2, 3; Supplementary Figure 2).

Conclusively, the efficacy observed in the combination group was better and not due to higher PD-L1 expression or less previous treatment.

### Distinct Baseline Metastases Have Differential Outcomes

**Figure 2:**
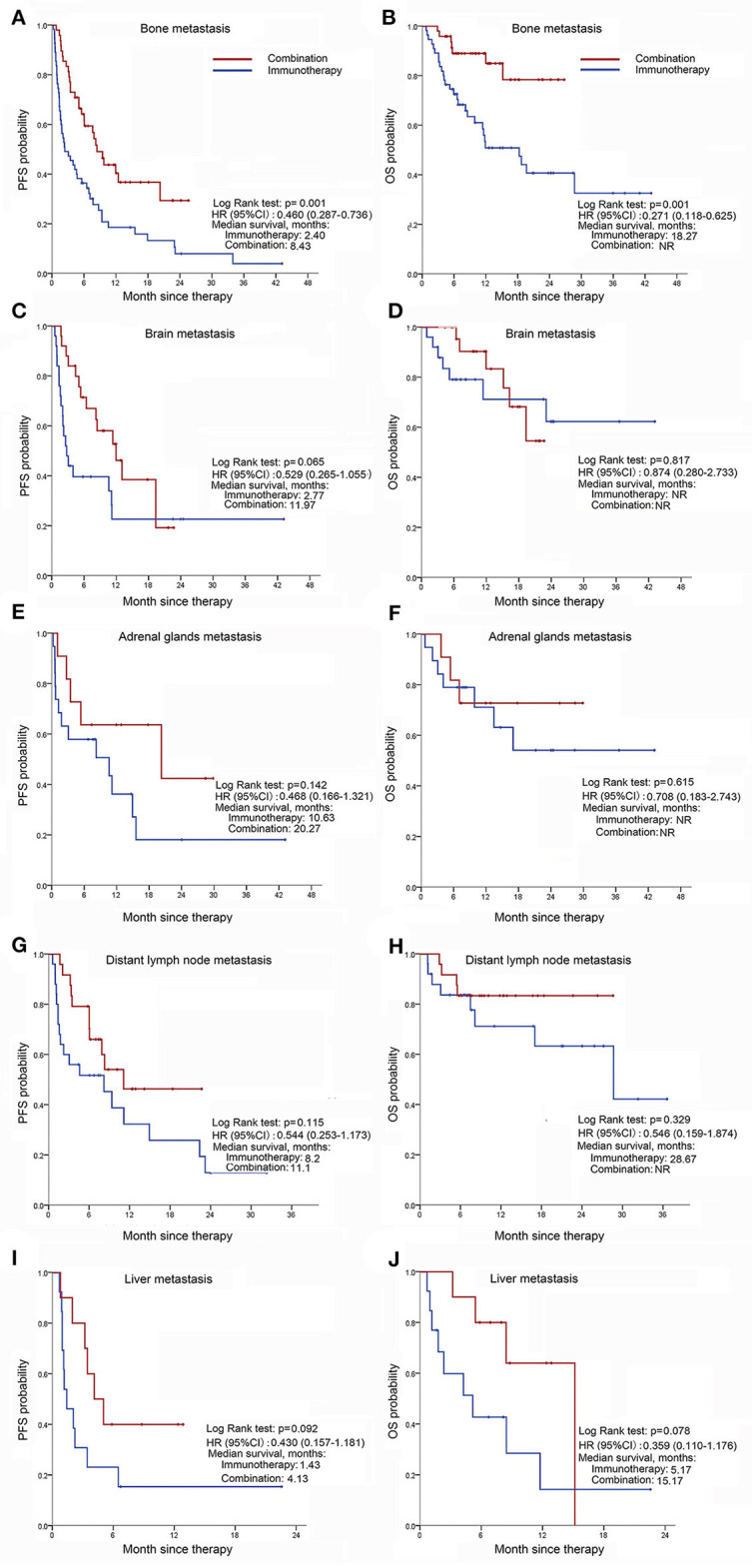
Distant metastases before immunotherapy associated with progression-free survival, and overall survival of tumors treated differently. **(A,B)** PFS **(A)** and OS **(B)** in patients with bone metastasis that had ICI monotherapy or in combination with chemotherapy; **(C,D)** PFS **(C)** and OS **(D)** in patients with brain metastasis that received immunotherapy alone or in combination with chemotherapy; **(E,F)** PFS **(E)** and OS **(F)** in patients with adrenal gland metastasis that had anti-PD-(L)1 antibody therapy or in combination with chemotherapeutics; **(G,H)** PFS **(G)** and OS **(H)** in patients with distant lymph node metastasis treated with ICI monotherapy or in combination with chemotherapy; **(I,J)** PFS **(I)** and OS **(J)** in patients with liver metastasis treated differently.

Given that metastasis is the dominant lethal event in NSCLC patients, most immunotherapy trials set stringent requirements for the eligible participants. Our study showed that, in patients with baseline bone metastases, the combination group provided an survival benefit when compared with monotherapy [median OS (95% CI): NR (NR–NR) vs. 18.3 (9.2–27.3), HR (95% CI): 0.271 (0.118–0.625); median PFS (95% CI): 8.4 (6.0–10.8) vs. 2.4 (0.2–4.6), HR (95% CI): 0.460 (0.287–0.736); Figures 2A,B]. Similar trend of PFS benefit was demonstrated in patients with liver, adrenal gland or distant lymph node metastases when received combination treatment though no statistically significant differences were found (Log Rank test); and no univariate benefit in OS was observed in those three subgroups. Considering the small number of patients with liver, adrenal gland or lymph node metastasis (23/325, 7.1%; 30/325, 9.2%; 49/325, 15.1%, respectively, Figures 2C–J), further research is warranted.

**Figure 3:**
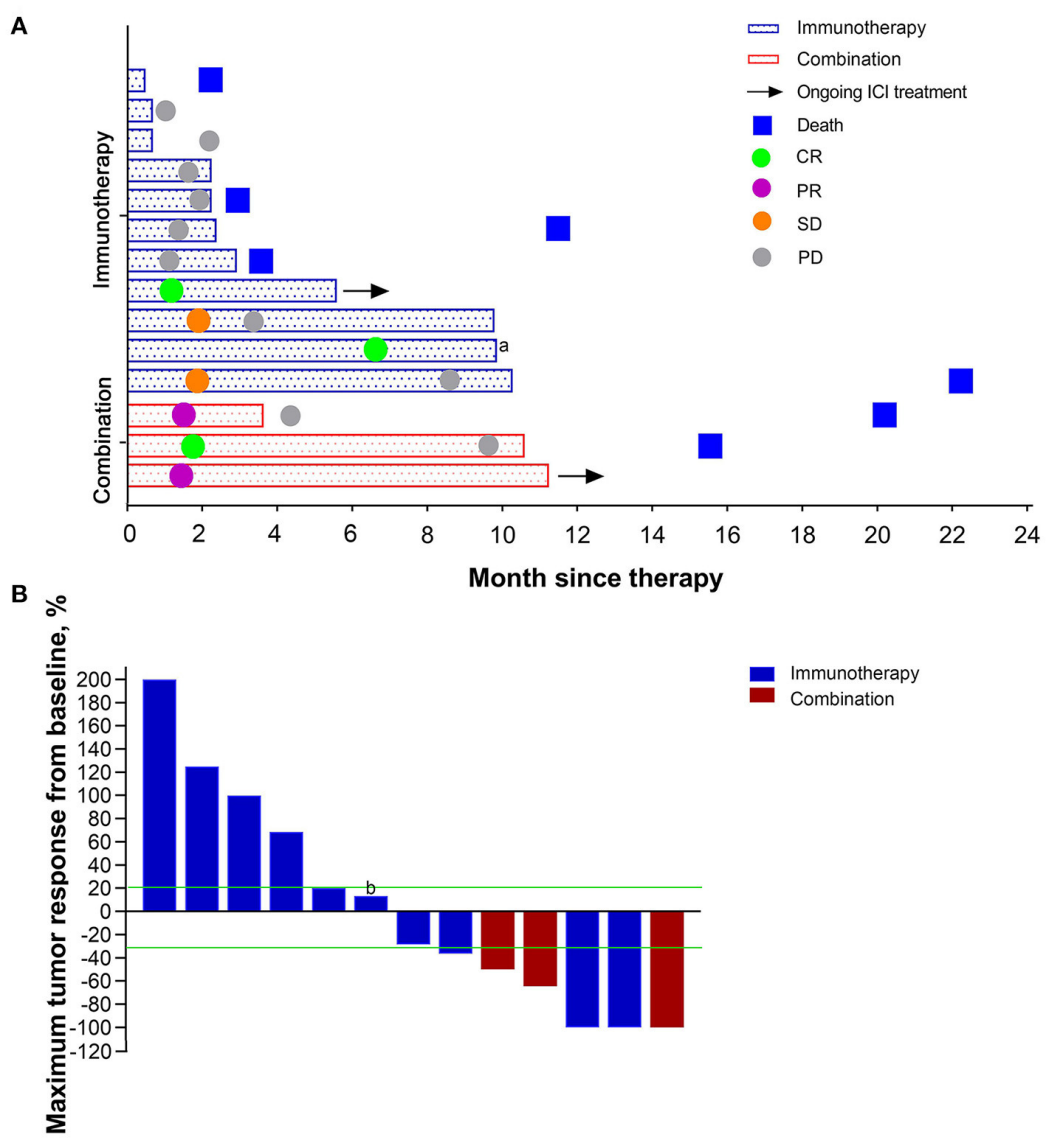
The responses and outcomes of patients with targeted brain metastases received immunotherapy alone or chemo-immunotherapy. **(A)** Time to brain metastasis response and duration of treatment. Bars represent individual patients who received immunotherapy. **(B)** Best brain metastasis response in assessable patients. The lower dashed line represents the −30% cut-off that defines an objective response. And the upper dashed line represents 20% cut-off that defines progression disease. CR, complete response; PR partial response; SD, stable disease; PD, progressive disease. ^a^One patient had developed progression of lung tumors and withdrew from immunotherapy despite 100% shrinkage of brain metastasis. ^b^One patient had progressive disease despite <20% enlargement due to the development of new brain metastasis.

The incidence of brain metastasis is apparently high in advanced NSCLC. The current standard regimen is becoming, early local therapies before or in conjunction with ICIs (13, 14). In order to evaluate the efficacy of immunotherapy, patients with target brain metastases (15) [without receiving whole-brain radiotherapy (WBRT) or stereotactic radiosurgery (SBS)] before immunotherapy were analyzed. The basic characteristics are shown in Table 3 and Figure 3A. Of patients with ICI alone, 2/11 (18.2%) had brain metastasis responses (CR; Figure 3B). The confirmed central nervous system (CNS) responses were durable (at data cutoff, responses had lasted 35.27, 4.23 months, respectively), but one patient discontinued ICI due to progression of pulmonary lesions. 2/11 (18.2%) patients had SD and 6/11 (54.5%) patients had PD in the CNS. 1/11 (9.1%) patient was unconfirmed in the CNS due to sudden death caused by rapid systemic progression. In contrast, all patients received combination treatment had brain lesion responses. The best response was CR in 1/3 (33.3%) patient. 2/3 (67.7%) patients had PR in the brain, although one patient had PD within half a year. Another patient with PR remained on treatment at data cutoff. In conclusion, combination ICI with chemotherapy has demonstrated a survival benefit for metastatic NSCLC patients.

**Table 3:** Baseline characteristics of patients with target brain metastases.

**Characteristic**	**Immunotherapy (*N* = 11)**	**Combination (*N* = 3)**
Age (mean ± SD, y)	63.27 ± 9.21	66.00 ± 8.19
Gender, *n* (%)		
Male	7 (63.6%)	3 (100%)
ECOG performance status, *n* (%)		
0–1	11 (100%)	3 (100%)
Tumor histology, *n* (%)		
Adenocarcinoma	9 (81.8%)	3 (100%)
Squamous cell carcinoma	1 (9.1%)	0 (0%)
NSCLC	0 (0%)	0 (0%)
Other	1 (9.1%)	0 (0%)
PD-L1 status, *n* (%)		
Known	6 (54.5%)	1 (33.3%)
Positive	5 (45.5%)	1 (33.3%)
≥25%	3 (27.3%)	1 (33.3%)
Numbers of target brain lesions per patient, mean ± SD	1.36 ± 0.924	1.33 ± 0.577
Total number of targeted lesions		
Previously untreated	15	4
Progressing after previous treatment	0	0
Size of all target lesions (mm)	158.35	30.23
Lines of ICIs, *n* (%)		
1	3 (27.3%)	3 (100%)
2	8 (72.7%)	0 (0%)
≥3	0 (0%)	0 (0%)

### Adverse Events and Outcomes

Notable trAEs that were in a higher incidence rate in the combination vs. immunotherapy group (≥1.5 times) were elevated transaminase or bilirubin, myelosuppression, electrolyte disturbance, ECG abnormalities, myalgia, dysfunction of intestine, constipation, nausea, and vomiting, hyperglycemia, pyrexia, alopecia, elevated creatinine and peripheral neuropathy (Table 4, Supplementary Table 5). Whereas, the incidence of hypothyroidism (≥1.5 times) was higher in immunotherapy arm. With these exceptions, the occurrence rates of dash, fatigue, hyperthyroidism, pneumonitis, decreased appetite, xerostomia, diarrhea, and hypertension were similar. Serious AEs (SAEs; stages 3–4) were reported in 10 (5.6%) patients from the immunotherapy group and 56 (38.1%) patients from the combination group (Supplementary Table 4). The SAEs were primarily related to myelosuppression in the Combination arm, of which the incidence rate was 32.7% (48/147). Of the SAEs that resulted in treatment delay (*n* = 17, 9.6%; *n* = 31, 21.1%, respectively), 12 (6.7%) and 20 (13.6%) patients were considered related to ICIs, respectively. Moreover, 12 (6.7%) patients in immunotherapy arm (7 interstitial lung disease cases, three hypothyroidism cases, one ICI-related encephalitis and one fatigue case) and 5 (3.4%) patients in combination arm (two interstitial lung disease cases, two dash cases, and one ICI-related myocarditis case) withdrew from treatment due to SAEs. Up to now, no treatment-related death was reported. Similar results were obtained in irAEs (Supplementary Table 6). All AEs were assessed by at least three independent medical professionals. Overall, no significant increase in the risk of irAEs was found after combination treatment.

**Table 4:** Incidence of treatment-related adverse events (trAEs)^a^.

**Events, *N* (%)**	**Immunotherapy (*****N*** **= 178)**					**Combination (*****N*** **= 147)**				
	**Total**	**Grade 1**	**Grade 2**	**Grade 3**	**Grade 4**	**Total**	**Grade 1**	**Grade 2**	**Grade 3**	**Grade 4**
Any adverse event	91 (51.1)					126 (85.7)				
Dash	34 (19.1)	27 (15.2)	6 (3.4)	1 (0.6)	0 (0)	33 (22.4)	24 (16.3)	3 (2.0)	4 (2.7)	2 (1.4)
Fatigue	30 (16.9)	27 (15.2)	0 (0)	2 (1.1)	0 (0)	33 (22.4)	28 (12.0)	5 (3.4)	0 (0)	0 (0)
Hyperthyroidism	20 (11.2)	19 (10.7)	1 (0.6)	0 (0)	0 (0)	13 (8.8)	13 (8.8)	0 (0)	0 (0)	0 (0)
Hypothyroidism	20 (11.2)	10 (5.6)	10 (5.6)	0 (0)	0 (0)	10 (6.8)	10 (6.8)	0 (0)	0 (0)	0 (0)
Elevated transaminase or bilirubin	16 (9.0)	15 (8.4)	0 (0)	1 (0.6)	0 (0)	39 (26.5)	34 (23.1)	2 (1.4)	2 (1.4)	1 (0.7)
Pneumonitis	15 (8.4)	2 (1.1)	10 (5.6)	1 (0.6)	2 (1.1)	15 (10.2)	5 (3.4)	7 (4.8)	3 (2.0)	0 (0)
Decreased appetite	11 (6.2)	11 (6.2)	0 (0)	0 (0)	0 (0)	9 (6.1)	8 (5.4)	1 (0.7)	0 (0)	0 (0)
Myelosuppression	10 (5.6)	5 (2.8)	3 (1.7)	2 (1.1)	0 (0)	106 (72.1)	22 (15.0)	36 (24.5)	33 (22.4)	15 (10.2)
Electrolyte disturbance	8 (4.5)	8 (4.5)	0 (0)	0 (0)	0 (0)	10 (6.8)	8 (5.4)	1 (0.7)	1 (0.7)	0 (0)
ECG abnormalities^b^	7 (3.9)	6 (3.4)	1 (0.6)	0 (0)	0 (0)	10 (6.8)	8 (5.4)	0 (0)	1 (0.7)	1 (0.7)
Myalgia	4 (2.2)	3 (1.7)	1 (0.6)	0 (0)	0 (0)	11 (7.5)	8 (5.4)	2 (1.4)	1 (0.7)	0 (0)
Constipation	3 (1.7)	3 (1.7)	0 (0)	0 (0)	0 (0)	6 (4.1)	5 (3.4)	1 (0.7)	0 (0)	0 (0)
Diarrhea	2 (1.1)	2 (1.1)	0 (0)	0 (0)	0 (0)	2 (1.4)	1 (0.7)	0 (0)	1 (0.7)	0 (0)
Nausea, vomiting	2 (1.1)	1 (0.6)	1 (0.6)	0 (0)	0 (0)	15 (10.2)	12 (8.2)	2 (1.4)	1 (0.7)	0 (0)
Hyperglycemia	2 (1.1)	2 (1.1)	0 (0)	0 (0)	0 (0)	13 (8.8)	13 (8.8)	0 (0)	0 (0)	0 (0)
Diarrhea	2 (1.1)	2 (1.1)	0 (0)	0 (0)	0 (0)	2 (1.4)	1 (0.7)	0 (0)	1 (0.7)	0 (0)
Hypertension	2 (1.1)	0 (0)	0 (0)	2 (1.1)	0 (0)	2 (1.1)	0 (0)	2 (1.1)	0 (0)	0 (0)
Myocarditis	0 (0)	0 (0)	0 (0)	0 (0)	0 (0)	1 (0.7)	0 (0)	0 (0)	1 (0.7)	0 (0)

a *The cutoff date was May 26, 2019; trAEs with an incidence of more than 5% are listed, and all grades 3–4 trAEs are listed*.

b *Arrhythmias, prolonged QT interval, inverted T wave, etc*.

*ECG, electrocardiogram*.

In our study, outcomes of patients with and without early irAEs (16) were shown in Supplementary Figure 3. The analysis showed that the development of early irAEs was significantly associated with increased PFS in immunotherapy arm [log-rank *P* = 0.053; multivariate HR (95% CI), 0.621 (0.411–0.941), *P* = 0.024], which were consistent with previous studies (16, 17). However, similar trend was not found in combination group (Supplementary Figures 3, 4).

**Figure 4:**
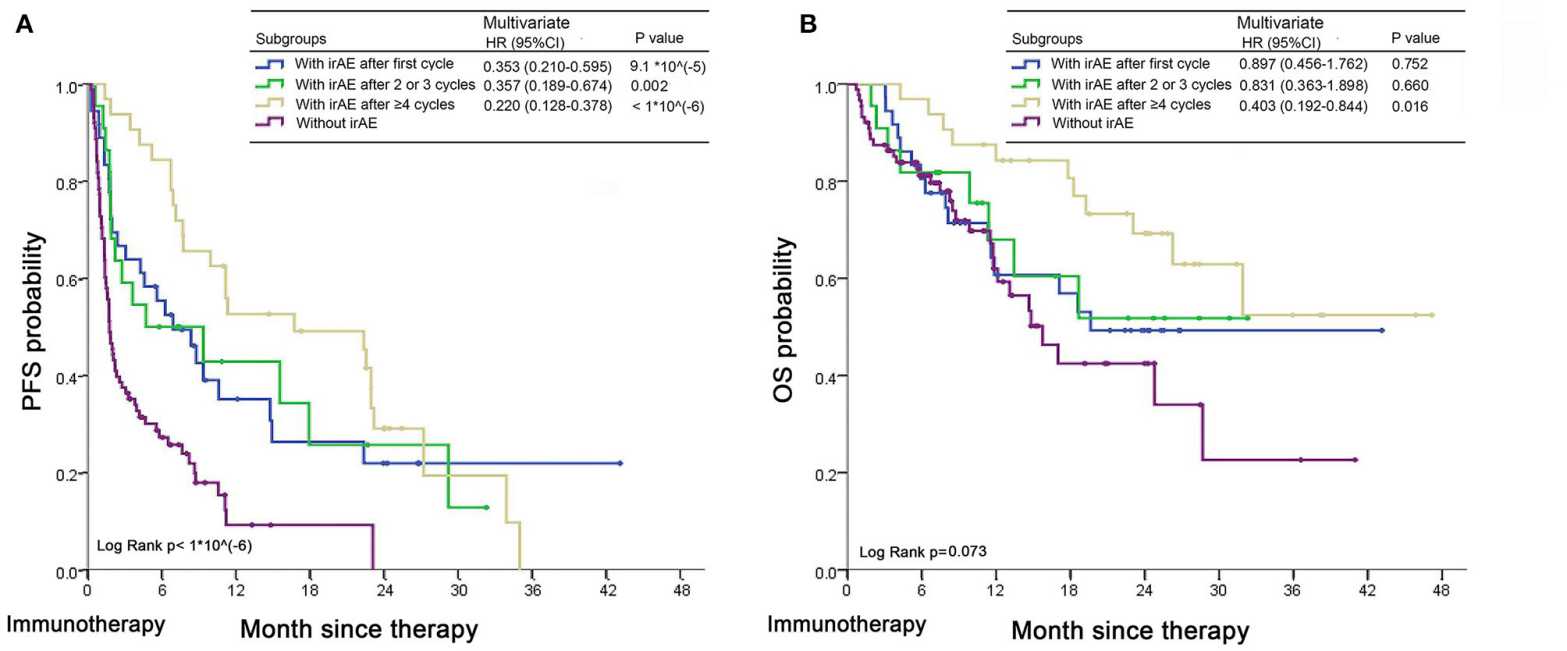
Time to onset irAEs and association with outcomes in advanced NSCLC patients treated with ICI monotherapy. **(A,B)** Kaplan-Meier curves of PFS **(A)** and OS **(B)** in patients with early (after 1–3 cycles), late (after four or more cycles) or without irAEs after commencement of ICI therapy. The multivariate HRs, 95% CIs and *P*-values were analyzed using a stratified Cox proportional hazards model taking into account gender (male, female), age (<65, ≥65), BMI (18) (<18.5, 18.5–22.9, 23–24.9, ≥25), smoking status (non-smoker, smoker), histology (adenocarcinoma, squamous carcinoma, NSCLC, neuroendocrine neoplasm, others), EGFR mutation (wild-type, mutation, unknown), PD-L1 expression level (<25%, ≥25%, unknown) and previous treatment lines (0, 1, ≥2). Log Rank *p*-values were calculated by log-rank test.

Patients with irAEs after 1 or 2–3 cycles of ICI-alone therapy had moderate prognosis; non-irAE predicted poorest outcome; while patients with late irAEs (≥4 cycles) had best outcomes [(irAEs after four or more cycles vs. non-irAEs) PFS: multivariate HR (95% CI), 0.220 (0.128–0.378), *p* <1^*^10^(−6)^; OS: multivariate HR (95% CI), 0.403 (0.192–0.844), *P* = 0.016] in the immunotherapy group (Figure 4). Conclusively, irAEs predicted better outcomes of immunotherapy, especially for patients with late irAEs.

## Discussion

In this real-world study, we assembled a cohort of 325 patients with advanced NSCLC treated with ICI to retrospectively investigate which treatment scenario is the most efficient: ICI monotherapy or in combination with chemotherapy, and how clinical factors impact response and survival of those patients. The combination group demonstrated promising anti-tumor activity and an acceptable side-effect profile regardless of PD-L1 level or previous treatment lines. Both regimens were well-tolerated, the more efficient is usually recommended.

Currently, there are many controversial issues regarding immunotherapy in the real-world practice. Firstly, the lack of direct cross-comparison studies in clinical trials between ICI monotherapy and chemo-immunotherapy. Secondly, the low detection rate and positive predictive value (19, 20) of PD-L1 or tumor mutation burden (TMB) in clinical practice of immunotherapy. Thirdly, ICI alone have demonstrated minimal benefit in liver metastases [a common metastasis and a negative prognostic indicator for lung cancer (21, 22)]. Some combination regimens were investigated in various randomized phase III studies (23, 24), but the final conclusion is still pending. Lastly, severe irAEs require high-dose intravenous steroids and even temporary or permanent discontinuation of ICIs (25). But the occurrence of irAEs was related to better outcomes in NSCLC subjects treated with ICIs (16, 17). How to balance between safety and efficacy of ICIs in the clinical practice? Our study has partially answered those issues.

Preclinical data have emerged suggesting that chemotherapy can significantly enhance the efficacy of certain forms of cancer immunotherapy (26). But the exact mechanism is still unclear. For one thing, studies have indicated that local chemotherapy combined with anti-PD-1 antibody facilitates an antitumor immune response and improves survival (*p* < 0.001) in glioblastoma, but addition of systemic chemotherapy to anti-PD-1 treatment resulted in systemic and intratumoral lymphodepletion, with decreased immune memory in long-term survivors (27); and the toxicity of some chemotherapeutic agents to immune cells limits the extent of immune stimulation and can lead to immunosuppression (28). For another, other researchers have demonstrated that certain conventional chemotherapies may have positive effects on tumor immunity: chemotherapy-induced immunogenic cell death activates innate immune responses and elicits a tumor-specific adaptive immune response (29, 30); it can directly block immunosuppressive pathways in the tumor microenvironment (TME) (31, 32).

Emerging data from clinical trials demonstrated that chemo-immunotherapy combination had better efficacy than ICI alone in certain clinical scenarios. We summarized these results in Supplementary Table 7. The survival benefits of combination treatment were significantly higher than ICI monotherapy. In the high PD-L1 cohort, the combinations of chemotherapy with ICIs were overall the better treatments regarding PFS; in the low PD-L1 arm (including negative), all combinations with chemotherapy examined (pembrolizumab, atezolizumab, nivolumab) showed superior outcomes to immunotherapy-alone regarding PFS and OS. Similar results were shown in our study, especially for patients with unknown PD-L1 status (195/325, 60%), which had longer PFS and OS in the combination group compared with the ICI monotherapy. When talking for the patients with PD-L1 ≥50%, it requires more prospective or external cohort data to further confirm whether combination treatment would be better as first-line treatment or not (Supplementary Table 2).

For most subgroups, the magnitude of treatment efficacy was greater in the combination arm than ICI-alone arm. Interestingly, we found that immunotherapy/chemotherapy seems to benefit more men than women [(median PFS, moths) monotherapy arm 6.27, 2.23, respectively, combination arm 15.53, 9.47, respectively]. However, different studies have different conclusions (33, 34). In addition, patients who smoke responded better to ICI combination therapies than non-smokers, which is consistent with previous studies (35, 36). And fundamental research had revealed that higher level of aryl hydrocarbon receptor (AhR) may play a role (37). BMI ≥25 was correlated with better outcomes of patients treated with ICI monotherapy according to previous studies (38), and our results also found that patients in the Combination arm had a similar trend (Supplementary Figure 2). Overweight could be considered a tumorigenic immune-dysfunction that could be effectively reversed by ICIs (39, 40). ICI combination treatment can improve outcomes in metastatic NSCLC patients according to our subgroup analyses, but a larger cohort of patients is necessary.

Safety is another primary concern. Our results revealed that patients with irAEs had longer PFS in ICI alone group, while no evidence showed that the occurrence of toxicity in the combination arm for patients is related to the efficacy. Previous studies have demonstrated that patients treated with immunotherapy with early irAEs had better outcomes (16, 17), nevertheless, other researches showed that the response of immunotherapy had no relationship with the side effects (41). Therefore, more research is needed on the relationship between efficacy and toxicity.

However, rational combination of chemotherapy and immunotherapy faces many challenges (42): the requirement for an accurate predictive biomarker of efficacy; optimization efficacy, safety and tolerability through appropriate drug ratios, dosing and scheduling; and possible combination approaches for patients who had low response rates after immunotherapy (such as EGFR mutations, liver metastasis and so on). The proportion of NSCLC patients with PD-L1 ≥50% was around 30% (43). In the real world, accurate expression level of PD-L1 is largely unknown (60%, our result). And the dynamics of PD-L1 expression may limit its use as a tissue-based predictive biomarker (44, 45). TMB also has many limitations (46, 47). To address the first challenge, we should make further efforts to deepen the mechanism study and careful design of biomarker exploratory studies. Recently, the number of ICIs and clinical trials of ICI combination treatment have growth rapidly. Different doses (36), sequencing possibilities (48, 49), combination therapy regimens (23, 50), and inclusion criteria (PD-L1 level, driver genes, PS ECOG and so on) (5, 9, 51) have different outcomes. Under the premise of ensuring safety, it is not an easy task to decide which regimen is best. Undoubtedly, it is unwise to simply increase the number of drugs in combination therapy. Comprehensive analysis of our study and clinical trials, the approach of ICI plus chemotherapy might have better anti-tumor activity than ICI monotherapy regardless of PD-L1 level in advanced NSCLC patients.

Our study has several limitations. Firstly, this research was a single-center retrospective study with a limited sample, inevitably, the AEs could be underreported due to the retrospective nature. The prospective or external cohort validations were needed to verify in the future. Secondly, there is 60% patients in this study without PD-L1 expression data. It is well-known that PD-L1 plays a crucial role in the progression of tumor by altering status of immune surveillance. KEYNOTE-024 study showed that pembrolizumab was associated with significantly longer PFS and OS, and is preferred as 1L treatment in advanced NSCLC with PD-L1 ≥50%. However, the ability of PD-L1 level to predict efficacy of immunotherapy in NSCLC is controversial (52, 53). TMB, neutrophil-to-lymphocyte ratio, MSI and some other biomarkers have also been reported to be predictive. Therefore, PD-L1 combined with other clinical biomarkers could benefit to predict the outcomes of immunotherapy. Thirdly, patients in the immunotherapy alone group at later line were relatively more, and patients in the immunotherapy combination group at first-line were relatively more, while this is the characteristic and real record of the real-world research. To balance the discrepancy and the multiple confounding factors, we did the cox proportional analysis in overall patients taking account into the number of treatment lines as a confounding factor. The results show that chemo-immunotherapy group had better PFS and OS in the multivariate analysis. Further univariate analysis among the first-line, second-line or later line, respectively repeated that combination treatment had better survival in each group (Supplementary Table 2). More prospective and external cohort validations are required to further confirm our results.

## Data Availability Statement

The raw data supporting the conclusions of this article will be made available by the authors, without undue reservation.

## Ethics Statement

The studies involving human participants were reviewed and approved by the Ethics Committee of Shanghai Chest Hospital. Written informed consent for participation was not required for this study in accordance with the national legislation and the institutional requirements.

## Author Contributions

XN and ZC: administrative support. XW, XN, and ZC: provision of study materials or patients. XW and XN: collection and assembly of data. XW, NA, and YS: data analysis and interpretation. All authors: conception, design, drafting manuscript, and final approval of manuscript.

## Conflict of Interest

The authors declare that the research was conducted in the absence of any commercial or financial relationships that could be construed as a potential conflict of interest.
