# Supplementary material for: Comparative Efficacy and Safety of Immunotherapy Alone and in Combination With Chemotherapy for Advanced Non-small Cell Lung Cancer
Source: Front Oncol. 2021 Mar 18;11:611012. doi: 10.3389/fonc.2021.611012 (PMC8013714; doi:10.3389/fonc.2021.611012)
Supplement: Supplementary file 1 [file Data_Sheet_1.docx]

Supplementary tables 1-7

Supplementary figures1-4

**Supplementary tables**

| **Supplementary Table 1** Therapeutic drugs | |
| --- | --- |
| Drugs | Dose |
| ICIs |  |
| Nivolumab | 3mg/kg·2W |
| Pembrolizumab | 200 mg/3W |
| Tislelizumab | 200 mg/3W |
| Sintilimab | 200 mg/3W |
| Camerelizumab | 200 mg/3W |
| Atezolizumab | 1200 mg/3W |
| Durvalumab | 20 mg/3W |
| Toripalimab | 3 mg/Kg·2W |
| Chemotherapeutics |  |
| Pemetrexed | 200 mg/m^2·3W |
| Paclitaxel | 175 mg/m^2·3W |
| Nab-Paclitaxel | 100 mg/m^2, d1, d8, d15 |
| Docetaxel | 75 mg/m^2·3W |
| Gemcitabine | 1250 mg/m^2, d1, d8 |
| Vinorelbine | 60 mg/m^2, d1, d8 |
| Etoposide | 100mg/ m^2·d, d1-d3 |

| **Supplementary Table 2** Previous treatment lines and survival | | | | | | | | | |
| --- | --- | --- | --- | --- | --- | --- | --- | --- | --- |
| Previous  treatment  line | Group | *N* | Median OS  95% CI | Log Rank *p* | Multivariate | Median PFS  95% CI | Log-  Rank *p* | Multivariate | |
|  |  |  |  |  | HR (95%CI), *P value* |  |  | HR (95%CI), | *P value* |
| 0 | IO | 33 | 17.0 (11.4-22.6) | 1*10^ (-6) | 0.188 (0.051-0.694), 0.012 | 5.8 (2.5-9.1) | 2*10^ (-6) | 0.275 (0.134-0.563), 0.000408 | |
|  | Combination | 114 | NR (NR-NR) |  |  | 19.4 (11.7-27.0) |  |  |  |
| 1 | IO | 109 | NR (NR-NR) | 0.187 | 0.362 (0.083-1.574), 0.175 | 5.6 (2.8-8.4) | 0.532 | 0.487 (0.237-0.997), 0.049 | |
|  | Combination | 19 | NR (NR-NR) |  |  | 6.9 [<1*10 ^ (-6)-14.7] |  |  |  |
| ≥ 2 | IO | 36 | NR (NR-NR) | 0.337 | 0.235 (0.031-1.787), 0.162 | 1.8 (1.6-2.1) | 0.024 | 0.179 (0.061-0.525), 0.002 | |
|  | Combination | 14 | NR (NR-NR) |  |  | NR (NR-NR) |  |  |  |

The HRs, 95% CIs and *p* values were determined using a stratiﬁed Cox proportional hazards model taking into account gender (male, female), age (< 65, ≥ 65), BMI (< 18.5, 18.5-22.9, 23-24.9, ≥ 25,unknown), smoking status (non-smoker, smoker), histology (adenocarcinoma, squamous carcinoma, NSCLC, neuroendocrine neoplasm, others), EGFR mutation (wild-type, mutation, unknown), and PD-L1 expression level (< 25%, ≥ 25%, unknown).

| **Supplementary Table 3** The expression level of PD-L1 and survival. | | | | | | | | | | |
| --- | --- | --- | --- | --- | --- | --- | --- | --- | --- | --- |
| PD-L1  TPS | Group | *N* | Median OS  95% CI | Log Rank *p* | Multivariate | | Median PFS  95% CI | Log Rank *p* | Multivariate | |
|  |  |  |  |  | HR (95%CI), *P value* | |  |  | HR (95%CI), *P value* | |
| < 25% | Immunotherapy | 34 | 14.7 (9.7-19.7) | 0.079 | 0.468 (0.130-2.686) | 0.246 | 2.1 (0.5-3.6) | 4.4*10^ (-4) | 0.547  (0.258-1.163) | 0.117 |
|  | Combination | 37 | NR (NR-NR) |  |  |  | 9.8 (NR-NR) |  |  |  |
| ≥ 25% | Immunotherapy | 33 | 17.0 (11.2-22.8) | 0.018 | 0.107  (0.020-0.581) | 0.010 | 5.8 (2.2-9.4) | 0.008 | 0.248 (0.097-0.631) | 0.003 |
|  | Combination | 26 | NR (NR-NR) |  |  |  | 20.3 (NR-NR) |  |  |  |
| Unknown | Immunotherapy | 111 | NR (NR-NR) | 0.001 | 0.154  (0.034-0.699) | 0.015 | 5.6 (1.6-9.6) | 7.5*10^ (-5) | 0.393 (0.188-0.821) | 0.013 |
|  | Combination | 84 | NR (NR-NR) |  |  |  | 15.5 (9.6-21.5) |  |  |  |

The HRs, 95% CIs and *p* values were determined using a stratiﬁed Cox proportional hazards model taking into account gender (male, female), age (< 65, ≥ 65), BMI (< 18.5, 18.5-22.9, 23-24.9, ≥ 25,unknown), smoking status (non-smoker, smoker), histology (adenocarcinoma, squamous carcinoma, NSCLC, neuroendocrine neoplasm, others), and previous treatment lines.

| **Supplementary Table 4** Characteristics of adverse events (AEs) | | | |
| --- | --- | --- | --- |
| Characteristics | Immunotherapy  (*N* = 178) | Combination  (*N* = 147) | *P* value |
| No. of patients with AEs, n (%) |  |  |  |
| trAE | 91 (51.1%) | 126 (85.7%) | < 1*10^ (-6) |
| irAE | 90 (50.6%) | 85 (57.8%) | 0.191 |
| Time to onset of AE, weeks, median (range) |  |  |  |
| trAE | 4.4 (0.1-74.4) | 1.6 (0.1-42.9) | 3.08*10^ (-4) |
| irAE | 4.4 (0.1-74.4) | 3.4 (0.1-74.6) | 0.736 |
| All grade 3–4 AEs |  |  |  |
| trAE | 10 (5.6%) | 56 (38.1%) | < 1*10^ (-6) |
| irAE | 8 (4.5%) | 10 (6.8%) | 0.365 |
| AEs leading to delayed treatment |  |  |  |
| trAE | 17 (9.6%) | 31 (21.1%) | 0.004 |
| irAE | 12 (6.7%) | 20 (13.6%) | 0.039 |
| AEs leading to withdrawal from treatment |  |  |  |
| trAE | 12 (6.7%) | 5 (3.4%) | 0.178 |
| irAE | 12 (6.7%) | 5 (3.4%) | 0.178 |
| Treatment-related death | 0 (0%) | 0 (0%) |  |

AEs were assessed by at least three independent medical professionals.

AEs, adverse events; trAEs, treatment-related adverse events; irAEs, immunodrug-related adverse events.

| **Supplementary Table 5** All of Treatment-Related Adverse Events (trAEs)^a^ | | | | | | | | | | | |
| --- | --- | --- | --- | --- | --- | --- | --- | --- | --- | --- | --- |
| Events, *N* (%) | Immunotherapy (*N*=178) | | | | | Combination (*N*=147) | | | | | |
|  | Total | Grade1 | Grade2 | Grade3 | Grade4 | Total | Grade1 | Grade2 | Grade3 | Grade4 | |
| Any adverse event | 91 (51.1) | | | | | 126 (85.7) |  |  |  |  |  |
| Dash | 34 (19.1) | 27 (15.2) | 6 (3.4) | 1 (0.6) | 0 (0) | 33 (22.4) | 24 (16.3) | 3 (2.0) | 4 (2.7) | 2 (1.4) |  |
| Fatigue | 30 (16.9) | 28 (15.7) | 0 (0) | 2 (1.1) | 0 (0) | 33 (22.4) | 28 (12.0) | 5 (3.4) | 0 (0) | 0 (0) |  |
| Hyperthyroidism | 20 (11.2) | 19 (10.7) | 1 (0.6) | 0 (0) | 0 (0) | 13 (8.8) | 13 (8.8) | 0 (0) | 0 (0) | 0 (0) |  |
| Hypothyroidism | 20 (11.2) | 10 (5.6) | 10 (5.6) | 0 (0) | 0 (0) | 10 (6.8) | 10 (6.8) | 0 (0) | 0 (0) | 0 (0) |  |
| Elevated transaminase or bilirubin | 16 (9.0) | 15 (8.4) | 0 (0) | 1 (0.6) | 0 (0) | 39 (26.5) | 34 (23.1) | 2 (1.4) | 2 (1.4) | 1 (0.7) |  |
| Pneumonitis | 15 (8.4) | 2 (1.1) | 10(5.6) | 1 (0.6) | 2 (1.1) | 15 (10.2) | 5 (3.4) | 7 (4.8) | 3 (2.0) | 0 (0) |  |
| Decreased appetite | 11 (6.2) | 11 (6.2) | 0 (0) | 0 (0) | 0 (0) | 9 (6.1) | 8 (5.4) | 1 (0.7) | 0 (0) | 0 (0) |  |
| Myelosuppression | 10 (5.6) | 5 (2.8) | 3 (1.7) | 2 (1.1) | 0 (0) | 106 (72.1) | 22 (15.0) | 36 (24.5) | 33(22.4) | 15(10.2) |  |
| Electrolyte disturbance | 8 (4.5) | 8 (4.5) | 0 (0) | 0 (0) | 0 (0) | 10 (6.8) | 8 (5.4) | 1 (0.7) | 1 (0.7) | 0 (0) |  |
| ECG abnormalities^b^ | 7 (3.9) | 6 (3.4) | 1 (0.6) | 0 (0) | 0 (0) | 10 (6.8) | 8 (5.4) | 0 (0) | 1 (0.7) | 1(0.7) |  |
| Myalgia | 4 (2.2) | 3 (1.7) | 1 (0.6) | 0(0) | 0(0) | 11 (7.5) | 8 (5.4) | 2 (1.4) | 1 (0.7) | 0 (0) |  |
| Constipation | 3 (1.7) | 3 (1.7) | 0 (0) | 0 (0) | 0 (0) | 6 (4.1) | 5 (3.4) | 1 (0.7) | 0 (0) | 0 (0) |  |
| Xerostomia | 2 (1.1) | 2 (1.1) | 0 (0) | 0 (0) | 0 (0) | 3 (2.0) | 2 (1.4) | 1 (0.7) | 0 (0) | 0 (0) |  |
| Nausea, vomiting | 2 (1.1) | 1 (0.6) | 1 (0.6) | 0 (0) | 0 (0) | 15 (10.2) | 12 (8.2) | 2 (1.4) | 1 (0.7) | 0 (0) |  |
| Hyperglycemia | 2 (1.1) | 2 (1.1) | 0 (0) | 0 (0) | 0 (0) | 13 (8.8) | 13 (8.8) | 0 (0) | 0 (0) | 0 (0) |  |
| Pyrexia | 2 (1.1) | 2 (1.1) | 0 (0) | 0 (0) | 0 (0) | 7 (4.8) | 6 (4.1) | 1 (0.7) | 0 (0) | 0 (0) |  |
| Alopecia | 2 (1.1) | 2 (1.1) | 0 (0) | 0 (0) | 0 (0) | 7 (4.8) | 7 (4.8) | 0 (0) | 0 (0) | 0 (0) |  |
| Elevated creatinine | 2 (1.1) | 2 (1.1) | 0 (0) | 0 (0) | 0 (0) | 5 (3.4) | 4(2.7) | 1(0.7) | 0 (0) | 0 (0) |  |
| Diarrhea | 2 (1.1) | 2 (1.1) | 0 (0) | 0 (0) | 0 (0) | 2 (1.4) | 1 (0.7) | 0 (0) | 1 (0.7) | 0 (0) |  |
| Hypertension | 2 (1.1) | 0 (0) | 0 (0) | 2 (1.1) | 0 (0) | 2 (1.4) | 0 (0) | 2 (1.4) | 0 (0) | 0 (0) |  |
| Edema | 2 (1.1) | 2 (1.1) | 0 (0) | 0 (0) | 0 (0) | 0 (0) | 0 (0) | 0 (0) | 0 (0) | 0 (0) |  |
| Encephalitis | 1 (0.6) | 0 (0) | 1 (0.6) | 0 (0) | 0 (0) | 0 (0) | 0 (0) | 0 (0) | 0 (0) | 0 (0) |  |
| Hemoptysis | 1 (0.6) | 0 (0) | 1 (0.6) | 0 (0) | 0 (0) | 0 (0) | 0 (0) | 0 (0) | 0 (0) | 0 (0) |  |
| Myocarditis | 0 (0) | 0 (0) | 0 (0) | 0 (0) | 0 (0) | 1 (0.7) | 0 (0) | 0 (0) | 1 (0.7) | 0 (0) |  |
| Peripheral neuropathy | 0 (0) | 0 (0) | 0 (0) | 0 (0) | 0 (0) | 5 (3.4) | 5 (3.4) | 0 (0) | 0 (0) | 0 (0) |  |

^a^ Data cutoff: 26 May, 2019

^b^ Arrhythmias, prolonged QT interval, inverted T wave, etc.

| **Supplementary Table 6** Immune-related Adverse Events (irAEs)^a^ | | |
| --- | --- | --- |
| Patients, *N* (%) | Immunotherapy (*N*=178) | Combination (*N*=147) |
| Any irAE  All grade  Grades 3-4 | 90 (50.6%)  8 (4.5%) | 85 (57.8%)  10 (6.8%) |
| Dash  All grades  Grades 3-4 | 34 (19.1)  1 (0.6) | 33 (22.4)  6 (4.1) |
| Pneumonitis  All grades  Grades 3-4 | 15 (8.4)  3 (1.7) | 15 (10.2)  3 (2.0) |
| Fatigue  All grades  Grades 3-4 | 30 (16.8)  3 (1.7) | 33 (22.4)  0 (0) |
| Hyperthyroidism  All grades  Grades 3-4 | 20 (11.2)  0 (0) | 13 (8.8)  0 (0) |
| Hypothyroidism  All grades  Grades 3-4 | 20 (11.2)  0 (0) | 10 (6.8)  0 (0) |
| Myelosuppression  All grades  Grades 3-4 | 9 (5.1)  2 (1.1) | 0 (0)  0 (0) |
| Alopecia  All grades Grades 3-4 | 2 (1.1)  0 (0) | 0 (0)  0 (0) |
| Hypertension  All grades  Grades 3-4 | 1 (0.6)  0 (0) | 2 (1.4)  0 (0) |
| Nausea, vomiting  All grades  Grades 3-4 | 2 (1.1)  0 (0) | 0 (0)  0 (0) |
| Decreased appetite  All grades  Grades 3-4 | 9 (6.2)  0 (0) | 9 (6.1)  0 (0) |
| ECG abnormalities^c^  All grades  Grades 3-4 | 7 (3.9)  0 (0) | 10 (6.8)  2 (1.4) |
| Constipation  All grades  Grades 3-4 | 3 (1.7)  0 (0) | 6 (4.1)  0 (0) |

| Diarrhea  All grades  Grades 3-4 | 2 (1.1)  0 (0) | 2 (1.4)  1 (0.7) |
| --- | --- | --- |
| Hyperglycemia  All grades  Grades 3-4 | 2 (1.1)  0 (0) | 10 (6.8)  0 (0) |
| Myalgia  All grades  Grades 3-4 | 4 (2.2)  0 (0) | 13 (8.8)  0 (0) |
| Myocarditis  All grades  Grades 3-4 | 0 (0)  0 (0) | 1 (0.7)  1 (0.7) |
| Encephalitis  All grades Grades 3-4 | 1 (0.6)  1 (0.6) | 0 (0)  0 (0) |
| Xerostomia  All grades Grades 3-4 | 3 (1.7)  0 (0) | 3 (2.0)  0 (0) |
| Electrolyte disturbance  All grades  Grades 3-4 | 8 (4.5)  0 (0) | 11 (7.5)  0 (0) |
| Pyrexia  All grades  Grades 3-4 | 0 (0)  0 (0) | 7 (4.8)  0 (0) |
| elevated creatinine  All grades  Grades 3-4 | 2 (1.1)  0 (0) | 5 (3.4)  0 (0) |

^a^ All irAEs were assessed by at least three independent medical professionals, and they were defined using MedDRA Preferred Terms that included both diagnosed immune conditions and signs and symptoms potentially representative of immune-related events^.^

| **Supplementary Table 7**  Clinical trials of immunotherapy in NSCLC patients | | | | | | | | | | | | |
| --- | --- | --- | --- | --- | --- | --- | --- | --- | --- | --- | --- | --- |
| Resectable | Study ID | | Drugs | *N* | Histology | Stage |  | | Primary endpoint | Neoadjuvant | Adjuvant | Year |
| Monotherapy | LCMC | | Atezolizumab | 84 | NSCLC | IB-IIIA |  | | MPR:19% | √ | √ | 2017-2024 |
|  | Checkmate-159 | | Nivolumab | 21 | NSCLC | IB-IIIA |  |  | MPR:19% | √ |  | 2019 |
| Combination | AAAQ3153 | | Atezolizumab/nTC | 18 | NSCLC | IB-IIIA |  | | MPR:50% | √ |  | 2016-2020 |
|  | Impower-030 | | Atezolizumab/Chemotherapy | 374 | NSCLC | IIA-IIIB |  |  | Ongoing | √ | √ | 2018-2024 |
|  | NEOSTAR | | Durvalumab/Chemotherapy | 44 | NSCLC | IA-IIIA |  |  | MPR:44% | √ |  | 2017-2022 |
|  | D9106C00001 | | Durvalumab/Chemotherapy | 300 | NSCLC | IIA-IIIB |  |  | Ongoing | √ | √ | 2018-2024 |
|  | Keynote-671 | | Pembrolizumab/Chemotherapy | 786 | NSCLC | IIB-IIIB |  |  | Ongoing | √ | √ | 2018-2026 |
|  | NADIM | | Nivolumab/TC | 46 | NSCLC | IIIA |  |  | PFS:85.36% | √ | √ | 2017-2022 |
|  | Checkmate-816 | | Nivolumab/Chemotherapy | 350 | NSCLC | IB-IIIA |  |  | Ongoing | √ |  | 2017-2028 |
|  | CA209-77T | | Nivolumab/Chemotherapy |  | NSCLC | IIA-IIIB |  |  | Ongoing | √ |  | 2019-2024 |
| Advanced | | Study ID | Drugs | *N* | Histology | Stage | PD-L1 | EGFR | Outcomes | Previous treatment lines | | Year |
| Monotherapy | GO28915 | | Atezolizumab | 425 | Non-Squamous NSCLC | IIIB/IV,  recurrent | All | WT | PFS: 2.8 (2.6-3.0)  OS: 13.8 (11.8-15.7)  ORR (%):13.6(10.53-17.28) |  | 1 | 2014-2019 |
|  | MYSTIC | | Durvalumab | 374 | NSCLC | IV | All | WT | PFS: 2.8 (2.6-3.1)  OS: 12.3 (10.1-14.9)  ORR (%): 22.2 |  | 0 | 2015-2020 |
|  | Keynote-024 | | Pembrolizumab | 154 | NSCLC | IV | ≥50% | WT | PFS: 10.3(6.7-NR)  OS: 30.0(18.3-NR)  ORR (%): 44.8(36.8- 53.0) |  | 0 | 2017-2021 |
|  | Keynote-042 | | Pembrolizumab | 638 | NSCLC | IV | ≥1% | WT | PFS: 5.4 (4.3-6.2)  OS: 16.7(13.9-19.7)  ORR (%): 27.3 (23.9-31.0) |  | 0 | 2018-2021 |
|  | Checkmate-026 | | Nivolumab | 271 | NSCLC | IV, recurrent | ≥1% | WT | PFS: 4.21 (3.06- 5.52)  OS: 13.73 (11.76-15.41)  ORR (%): 26.1 (20.3-32.5) |  | 0 | 2014-2020 |
|  | YO29232 | | Atezolizumab |  | NSCLC | IIIB/IV | ≥1% | All | Ongoing |  | ≥1 | 2016-2021 |
|  | D419AC00002 | | Durvalumab |  | NSCLC | IV | ≥25% | WT | Ongoing |  | 0 | 2016-2021 |
|  | MS200647_0037 | | M7824 or  Pembrolizumab | 584 | NSCLC | IV | High | WT | Ongoing |  | 0 | 2018-2024 |
|  | CA209-870 | | Nivolumab | 400 | NSCLC | IIIB/IV | All | All | Ongoing |  | 1 or 2 | 2017-2022 |
|  | CA209-227 | | Nivolumab |  | NSCLC | IV, recurrent | All | WT | Ongoing |  | 0 | 2018-2022 |
| Combination | IMpower-130 | | Atezolizumab/nTC | 451 | Non-Squamous NSCLC | IV | All | WT | PFS: 7.0 (6.2 - 7.3)  OS: 18.6 (16.0 - 21.2)  ORR (%): 49.2 |  | 0 | 2015-2020 |
|  | IMpower-131 | | Atezolizumab/TC | 338 | Squamous NSCLC | IV | All | WT | PFS: 5.6 (5.5 - 6.9)  OS: 12.6 (11.6 - 14.7) |  | 0 | 2015-2020 |
|  |  | | Atezolizumab/nTC | 343 | Squamous NSCLC | IV | All | WT | PFS: 6.5 (5.7 - 7.1)  OS: 14.2 (12.3 - 16.8) |  | 0 | 2015-2020 |
|  | Keynote-189 | | Pembrolizumab/AC | 410 | NSCLC | IV | All | WT | PFS: 8.8(7.6 - 9.2)  OS: NR (NR-NR)  ORR (%)^a^: 47.6 (42.6-52.5) |  | 0 | 2015-2020 |
|  | Keynote-407 | | Pembrolizumab/nTC or TC | 278 | NSCLC | IV | All | WT | PFS: 6.4 (6.2 - 8.3)  OS: 15.9 (13.2 - NR)  ORR (%): 57.9 (51.9 - 63.8) |  | 0 | 2016-2020 |
|  | IMpower-132 | | Atezolizumab/AC | 292 | Non-Squamous NSCLC | IV | All | WT | PFS: 7.6 (6.6-8.5)  OS: 18.1 (13.0-NR)  ORR (%):47 |  | 0 | 2016-2022 |
|  | Keynote789 | | Pembrolizumab/AC |  | Non-Squamous NSCLC | IV | All | MU | Ongoing |  | ≥1 | 2018-2023 |
|  | BGB-A317-304 | | Tislelizumab/AC |  | Non-Squamous NSCLC | IV | All | WT | Ongoing |  | 0 | 2018-2020 |
|  | BGB-A317-307 | | Tislelizumab/TC |  | Squamous NSCLC | IV | All | WT | Ongoing |  | 0 | 2018-2020 |
|  | CA209-722 | | Nivolumab/AC |  | NSCLC | IV, recurrent | All | MU^b^ | Ongoing |  | ≥1 | 2016-2025 |
|  | CA209-227 | | Nivolumab/Chemotherapy |  | NSCLC | IV, recurrent | All | WT | Ongoing |  | 0 | 2018-2022 |

^a^Overall response rate; ^b^without T790M mutation.

nTC, nab-paclitaxel + platinum; TC, paclitaxel + platinum; AC, pemetrexed + platinum.

**Supplementary figures**

**Supplementary Figure 1**

**A.**

B.

Supplementary Figure 1. Forest plot showing results of subgroup analysis of outcomes.

The overall HRs, 95%CIs and *p* values of PFS (A and OS (B) were analyzed using a stratiﬁed Cox proportional hazards model taking into account gender (male, female), age (< 65, ≥ 65), BMI (< 18.5, 18.5-22.9, 23-24.9, ≥ 25, unknown), smoking status (non-smoker, smoker), EGFR mutation (wild-type, mutation, unknown), histology (adenocarcinoma, squamous carcinoma, NSCLC, neuroendocrine neoplasm, others), PD-L1 expression level (< 25%, ≥ 25%, unknown), previous treatment lines (0, 1, ≥ 2) and distant metastasis (bone, brain, adrenal gland, lymph node, liver). And subgroup analyses were done with unstratified HRs estimated from a Cox proportional hazards model.

**Supplementary Figure 2**

A.


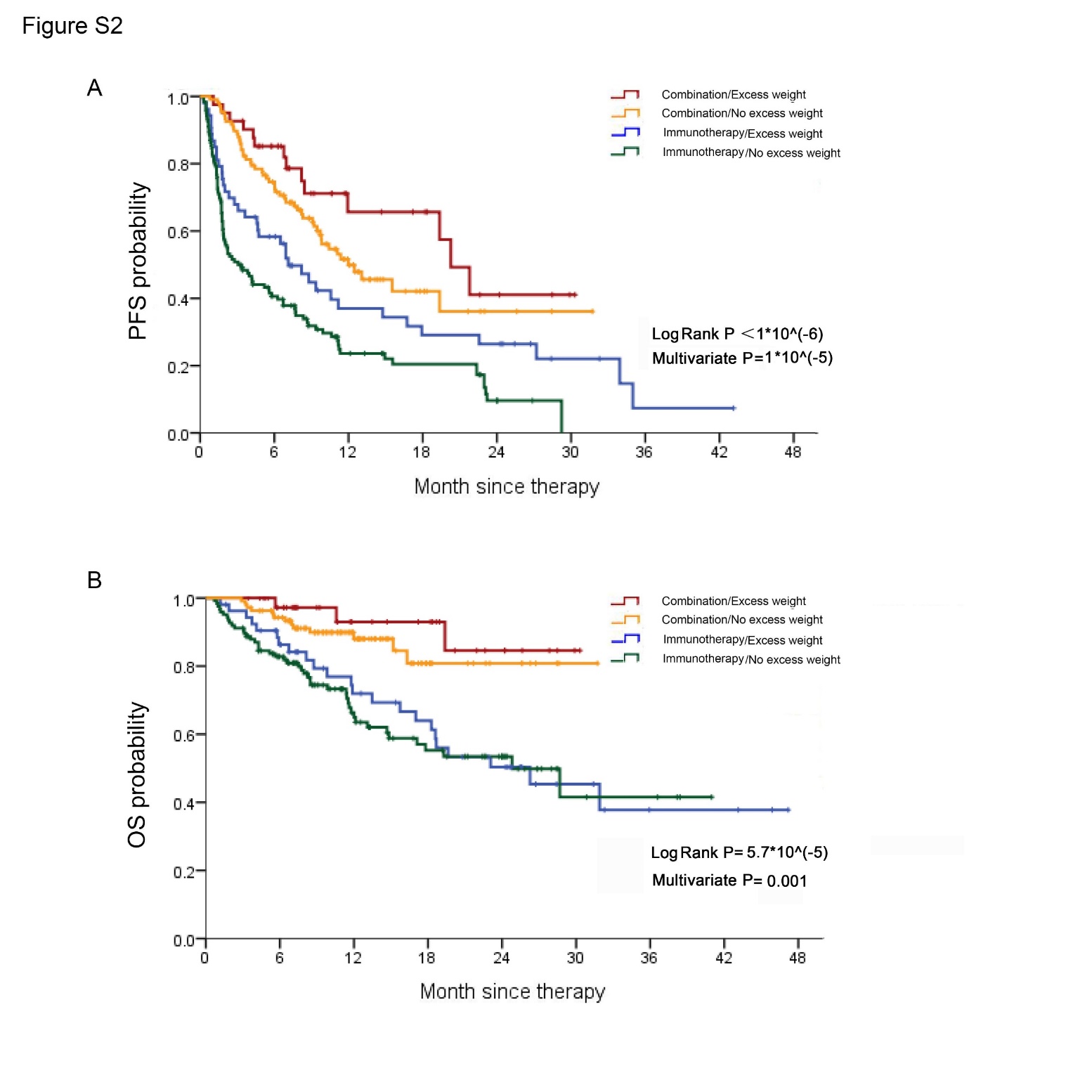


B.


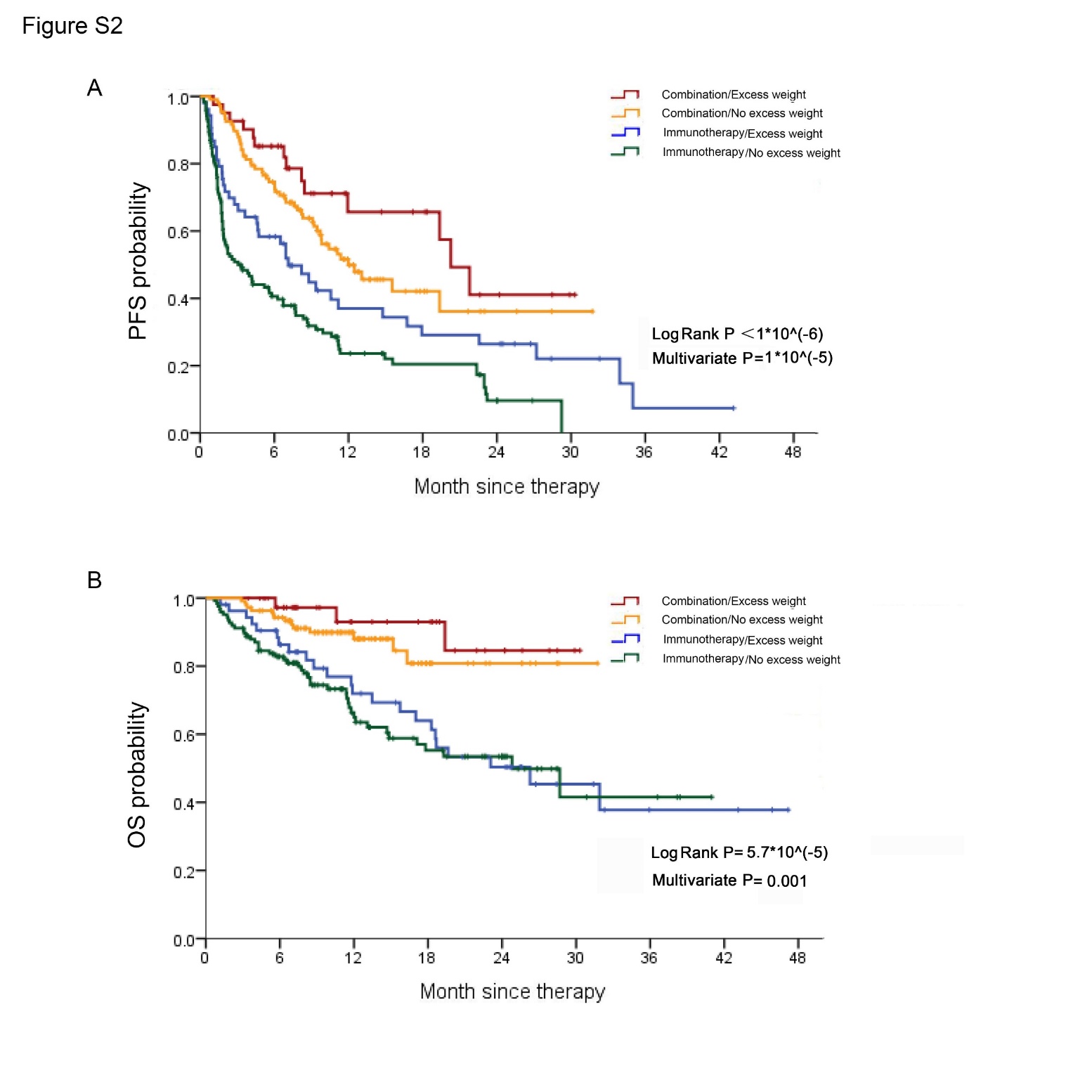


Supplementary Figure 2. Association between outcomes and BMI for ICI- or combination- treated patients.

(A) PFS and (B) OS for patients (non-excess or excess weight) treated differently.

The multivariate *p* values were analyzed using a stratiﬁed Cox proportional hazards model taking into account gender (male, female), age (< 65, ≥ 65), smoking status (non-smoker, smoker), histology (adenocarcinoma, squamous carcinoma, NSCLC, neuroendocrine neoplasm, others), EGFR mutation (wild-type, mutation, unknown), PD-L1 expression level (< 25%, ≥ 25%, unknown) and previous treatment lines (0, 1, ≥ 2). Log Rank *p* was calculated by log-rank test.

**Supplementary Figure 3.**

A. B.


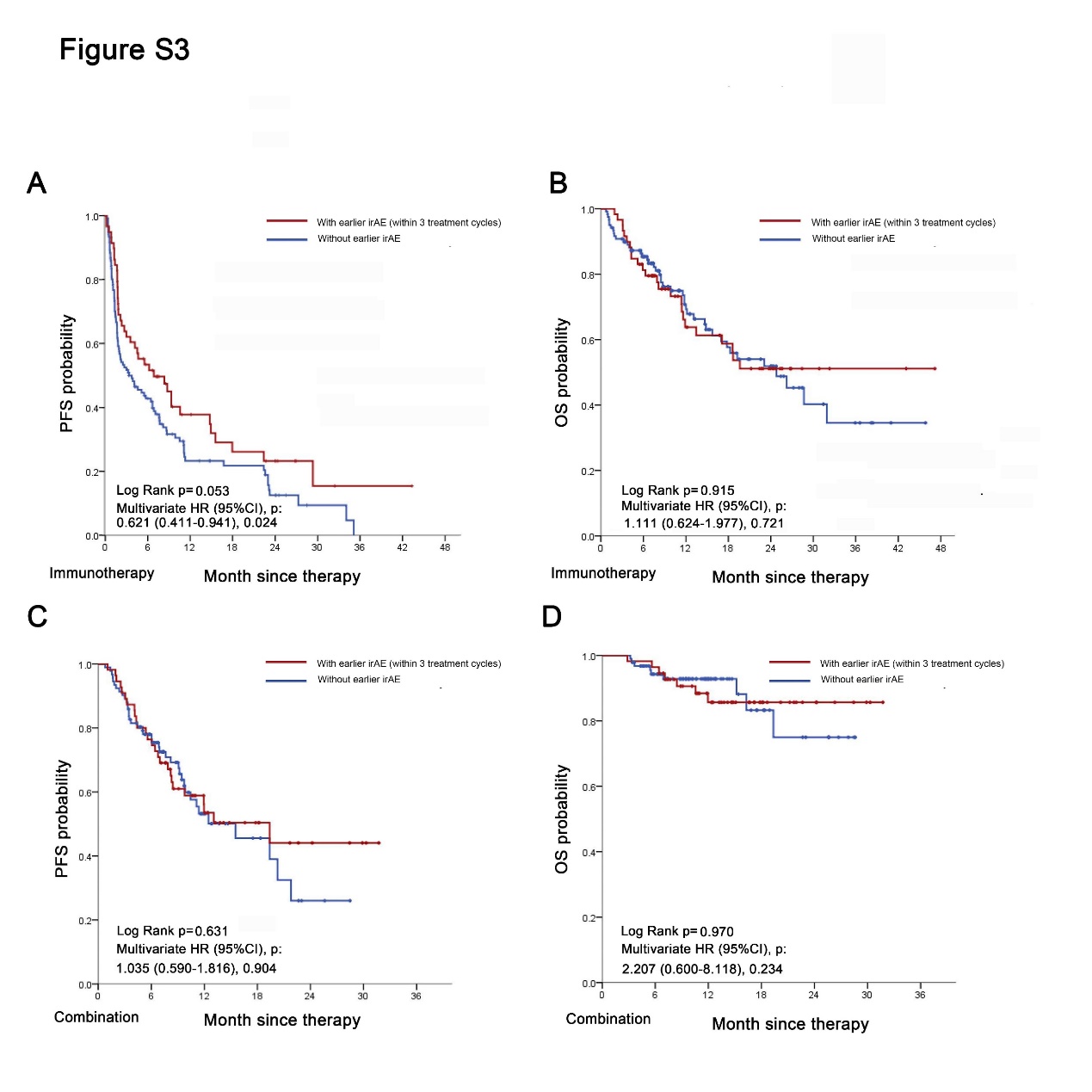


C. D.


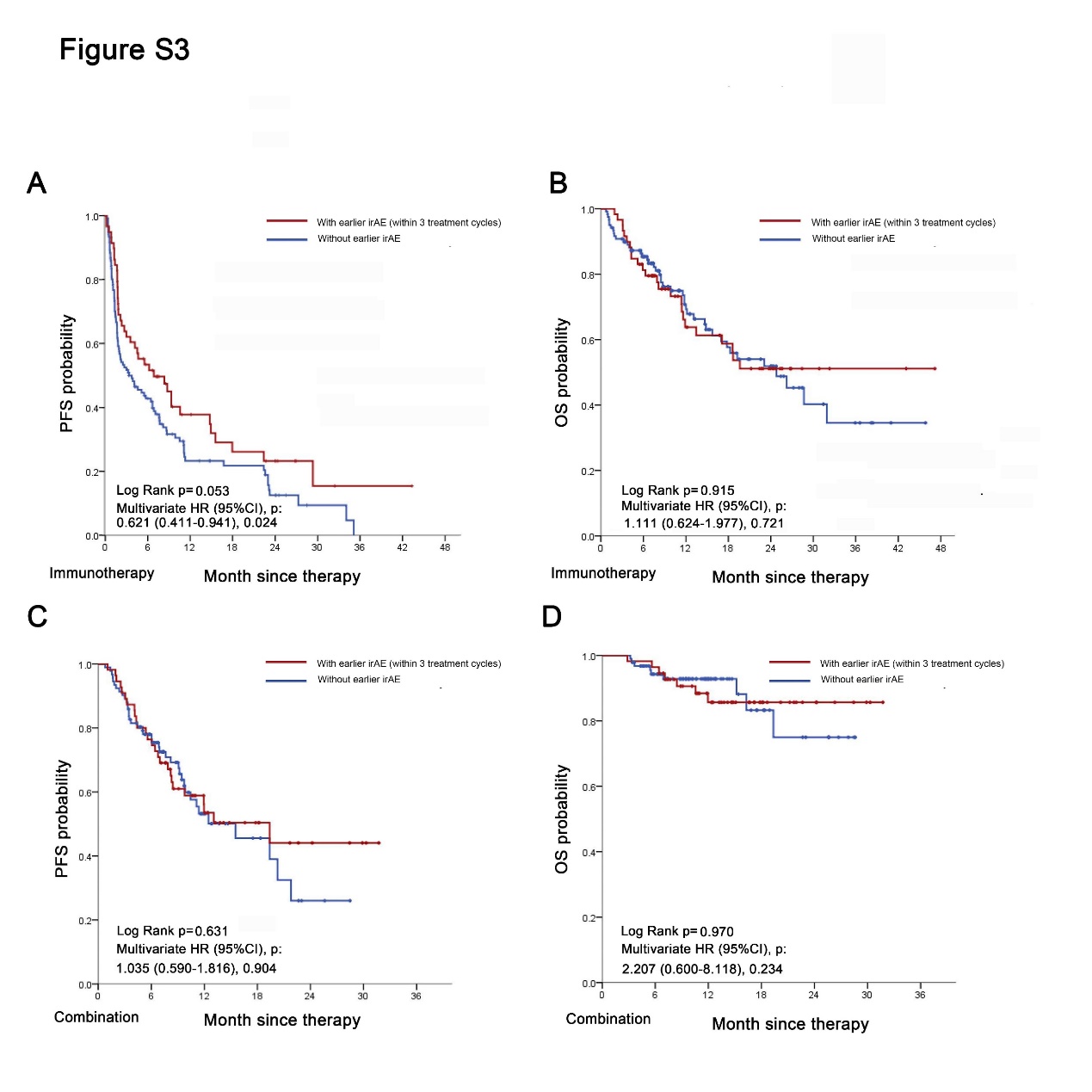


Supplementary Figure 3. Kaplan-Meier curves of PFS and OS in patients with or without earlier irAEs (within 3 cycles) after commencement of immunotherapy alone (A, B) or chemo-immunotherapy (C, D).

The multivariate HRs, 95%CIs and *p* values were analyzed using a stratiﬁed Cox proportional hazards model taking into account gender (male, female), age (< 65, ≥ 65), BMI (< 18.5, 18.5-22.9, 23-24.9, ≥ 25), smoking status (non-smoker, smoker), histology (adenocarcinoma, squamous carcinoma, NSCLC, neuroendocrine neoplasm, others), EGFR mutation (wild-type, mutation, unknown), PD-L1 expression level (< 25%, ≥ 25%, unknown), previous treatment lines (0, 1, ≥ 2) and distant metastasis (bone, brain, adrenal gland, lymph node, liver). Log Rank *p* was calculated by log-rank test.

**Supplementary Figure 4**

A. B.


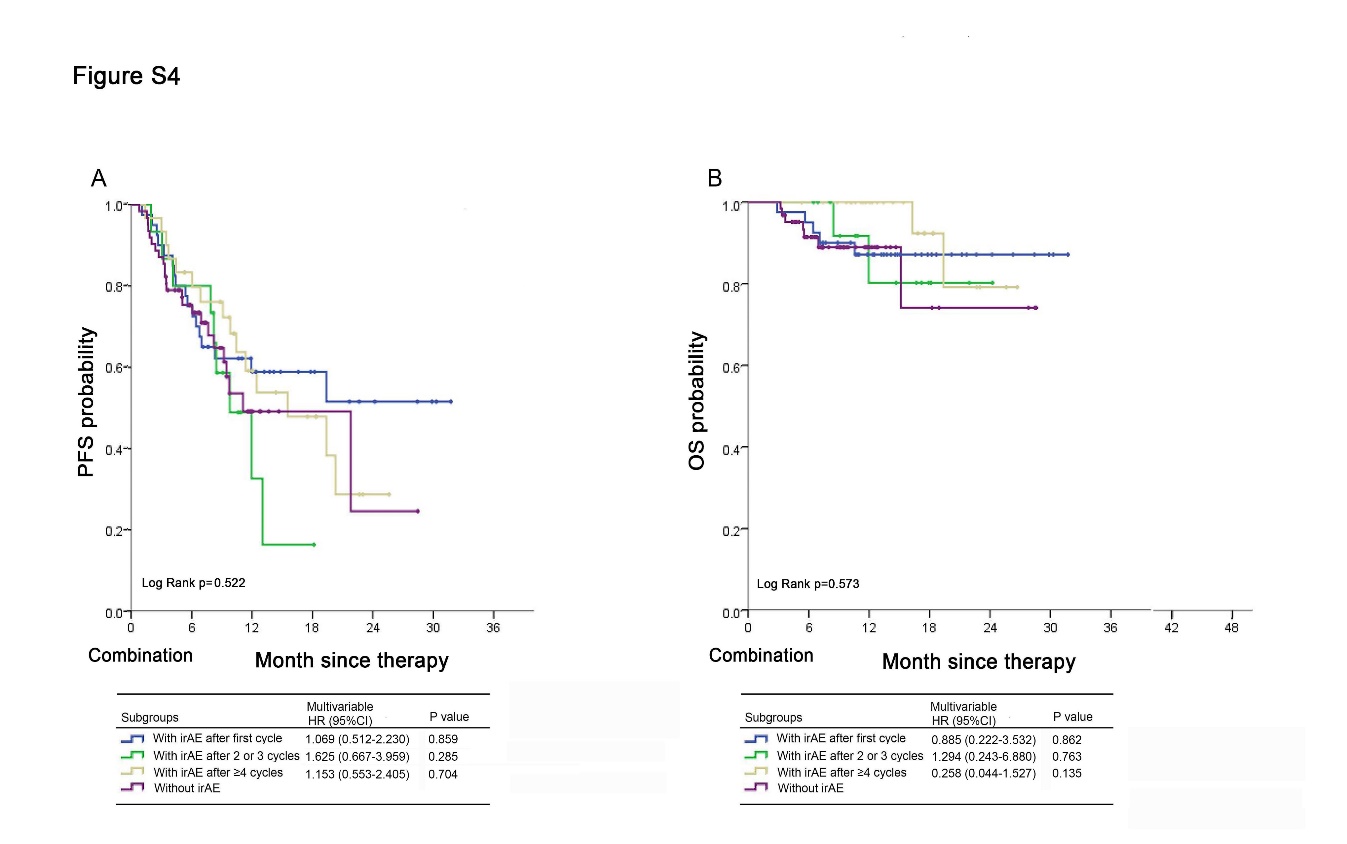


Supplementary Figure 4. Time to onset irAEs and association with outcomes in advanced NSCLC patients treated with chemo-immunotherapy.

(A) Kaplan-Meier curves of PFS and OS (B) in patients with early (after 1-3 cycles), late (after 4 or more cycles) or without irAEs after commencement of immunotherapy.

The multivariate HRs, 95%CIs and *p* values were analyzed using a stratiﬁed Cox proportional hazards model taking into account gender (male, female), age (< 65, ≥ 65), BMI (< 18.5, 18.5-22.9, 23-24.9, ≥ 25, unknown), smoking status (non-smoker, smoker), histology (adenocarcinoma, squamous carcinoma, NSCLC, neuroendocrine neoplasm, others), EGFR mutation (wild-type, mutation, unknown), PD-L1 expression level (< 25%, ≥ 25%, unknown) and previous treatment lines (0, 1, ≥ 2). Log Rank *p* values were calculated by log-rank test.
